# Autoimmune Pancreatitis Presenting as Obstructive Jaundice Mimicking Pancreatic Cancer: A Case Report

**DOI:** 10.7759/cureus.37947

**Published:** 2023-04-21

**Authors:** Tarun Kumar Suvvari, Sai Tejeswi Godavari, Praveen Sanapala, Smruthi Panchagnula, Sri Kruthi Alaka Nandha Godavari

**Affiliations:** 1 Research, Squad Medicine and Research (SMR), Visakhapatnam, IND; 2 General Medicine, Rangaraya Medical College, Kakinada, IND; 3 Internal Medicine, Ganni Subba Lakshmi (GSL) Medical College, Rajahmundry, IND; 4 Internal Medicine, Rangaraya Medical College, Kakinada, IND

**Keywords:** potential pitfall for misdiagnosis, autoimmune pancreatitis (aip), case report, misdiagnosis, pancreatic cancer

## Abstract

Autoimmune pancreatitis (AIP) is a rare form of chronic pancreatitis that can be misdiagnosed as pancreatic cancer due to similar clinical and radiological findings. In this case report, we present a 49-year-old male patient who presented with obstructive jaundice and was initially diagnosed with pancreatic cancer based on imaging findings. However, the lack of definitive parenchymal tissue in the biopsy raised suspicion for an alternative diagnosis, which led to further testing and ultimately the diagnosis of AIP. The use of endoscopic ultrasonography (EUS) and fine needle biopsy (FNB) helped to obtain a tissue diagnosis and rule out malignancy. The measurement of serum IgG4 levels further supported the diagnosis of AIP. The patient was treated with glucocorticoids and showed gradual improvement, ultimately recovering from AIP. This case highlights the importance of maintaining a high level of suspicion and considering AIP as a possible diagnosis when investigating cases that mimic pancreatic cancer. Early recognition and treatment with steroids can result in a favorable outcome for patients with AIP.

## Introduction

Autoimmune pancreatitis (AIP) is a chronic fibroinflammatory disorder of the pancreas, characterized by a dense lymphoplasmacytic infiltrate with fibrosis, which can lead to pancreatic exocrine and/or endocrine dysfunction [1]. The underlying pathogenesis of AIP involves an autoimmune response, whereby immune cells infiltrate and attack pancreatic tissue, resulting in progressive inflammation and fibrosis [2]. AIP is a rare disease with a reported incidence of less than 1% of cases per 100,000 population per year [3]. It is more common in males and typically presents in middle-aged to older adults [3]. AIP is often misdiagnosed as pancreatic cancer due to its similar clinical and radiological presentation [3]. AIP and pancreatic cancer have similar clinical symptoms that include abdominal pain, unexplained weight loss, obstructive jaundice, and fatigue. AIP can present radiographically as a focal mass that is similar to pancreatic cancer [2,3]. The international consensus diagnostic criteria (ICDC) for AIP were established in 2011 to aid in the diagnosis of AIP [3]. The criteria include clinical, radiological, histological, and serological parameters. The ICDC also distinguishes between two subtypes of AIP: type 1, which is associated with elevated serum IgG4 levels and extra-pancreatic manifestations, and type 2, which is IgG4-negative and may present with granulocytic epithelial lesions (GEL) on histology [3,4]. We present a case of AIP presenting as obstructive jaundice that mimicked pancreatic cancer.

## Case presentation

**Figure 1:**
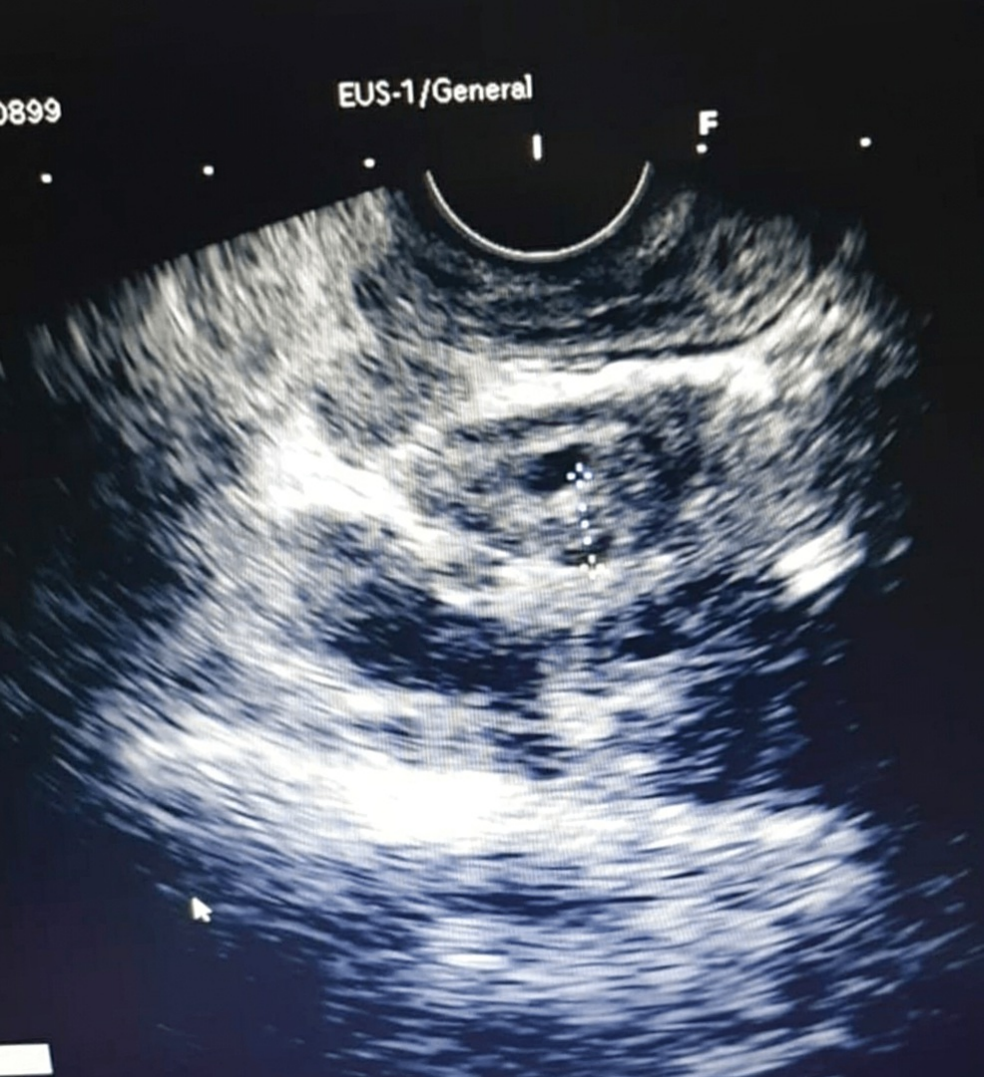
EUS showing CBD that is non-dilated (3 mm) but thick walled EUS, endoscopic ultrasonography; CBD, common bile duct

A 49-year-old male patient presented with obstructive jaundice of six months duration at a local hospital. At presentation, the patient was asymptomatic with no icterus, and the liver function tests were normal. He had h/o weight loss and appetite for four months. The patient did not have any known risk factors or a history of autoimmune disorders and no history of similar complaints in the past. No history of CECT of abdomen revealed a mass lesion at the head of the pancreas. Stenting was done to the main pancreatic duct (MPD) to relieve jaundice. A biopsy from the lesion at the head of the pancreas was done. Histopathology reveals fibrinous material and blood clots with scattered neutrophils and lymphocytes without parenchymal tissue, very thin fibrocollagenous tissue with crushing artifact with few neutrophils and lymphocytes. Occasional plasma cells that are negative for igG4 are noted. On endoscopic ultrasonography (EUS), pancreatic parenchyma appears bulky and hypoechoic with few hyperechoic strands; MPD is not dilated with a stent in situ. Multiple lymph nodes are present and the largest one (2x1.5 cm) is noted in the periportal region (benign appearing). EUS-guided fine needle biopsy (FNB) was done from the pancreatic head region and peripheral node. Common bile duct (CBD) is non-dilated (3 mm) but thick walled (Figure 1). EUS shows a bulky hypoechoic pancreas with periportal lymph nodes and thick-walled non-dilated CBD. Endoscopic retrograde cholangiopancreatography (ERCP) of pancreatic duct stent removal was done. Serum IgG4 levels were elevated (31.50 gm/dl) and a diagnosis of AIP was made. Glucocorticoids (steroid therapy) were administered to the patient and serum IgG4 levels were monitored. Prednisone (5 mg/day) was given for four weeks and further tapered to 2.5 mg/day for two weeks. The patient was followed up and showed gradual improvement, ultimately recovering from AIP.

## Discussion

AIP is a challenging diagnosis that requires careful consideration of the patient's clinical presentation, imaging findings, and laboratory results. When evaluating a patient with suspected AIP, it is important to consider the differential diagnosis, as the clinical presentation of AIP can overlap with other pancreatic disorders. Differential diagnoses for pancreatic lesions include other types of chronic pancreatitis and pancreatic cancer. Other types of chronic pancreatitis can present with similar symptoms and laboratory abnormalities to those seen in AIP. These can include alcoholic pancreatitis, hereditary pancreatitis, and idiopathic pancreatitis. In cases of alcoholic pancreatitis, the patient may have a history of heavy alcohol use, which can help differentiate it from AIP. Hereditary pancreatitis may be suspected if the patient has a family history of pancreatic disease. Idiopathic pancreatitis is a diagnosis of exclusion and is used when no other cause of pancreatitis can be identified [5,6]. AIP is a rare form of chronic pancreatitis that is often misdiagnosed as pancreatic cancer due to similar clinical and radiological findings [5,7]. The diagnosis of AIP is based on a combination of clinical, serological, and imaging findings. The normal range for serum IgG4 levels is 0.86-1.35 g/L, and in pancreatic cancer, it typically remains within this range. However, elevated serum IgG4 levels are a useful biomarker for AIP, with a sensitivity of around 60%-80% and a specificity of more than 90% [2]. The use of EUS and FNB has also shown to be valuable in obtaining a tissue diagnosis and ruling out malignancy [6,7].

The treatment for AIP usually involves a course of corticosteroids (steroid therapy). The dose and duration of steroid therapy can vary but recommended dose of prednisone is 0.4-0.6 mg/kg/day for one to two months, followed by a tapering schedule for a few months [8]. Some studies have suggested that a lower starting dose of prednisone (e.g., 0.5 mg/kg/day) may be effective in inducing remission while reducing the risk of steroid-related adverse effects. In cases where steroid therapy is ineffective or if the patient experiences disease relapse, alternative treatment options may include immunosuppressive agents, such as azathioprine, mycophenolate mofetil, or rituximab [8.9]. Biliary drainage procedures may also be necessary in cases where obstructive jaundice is present. The long-term prognosis for AIP patients is generally favorable, with most patients achieving and maintaining remission with appropriate treatment. However, disease relapse can occur in up to 50% of patients, and long-term monitoring is, therefore, recommended [9]. 

In our case, the patient underwent stenting of the MPD to relieve jaundice, and a biopsy was taken from the lesion. The histopathology of the biopsy showed fibrinous material and blood clots with scattered neutrophils and lymphocytes without parenchymal tissue, which is not typical of pancreatic cancer. However, imaging findings, including a mass lesion at the head of the pancreas and periportal lymph nodes, were suggestive of pancreatic cancer. The use of EUS and FNB helped to obtain a tissue diagnosis and rule out malignancy. EUS further revealed a bulky, hypoechoic pancreas with a few hyperechoic strands and a thick-walled but non-dilated CBD. The lack of definitive parenchymal tissue in the biopsy raised suspicion for an alternative diagnosis, which led to further testing and ultimately the diagnosis of AIP. The measurement of serum IgG4 levels further supported the diagnosis of AIP. Early recognition and treatment with steroids can result in a favorable outcome for patients with AIP [4,5]. A limitation of this approach is that the diagnosis of AIP can be challenging and requires a high degree of suspicion. In addition, the clinical presentation of AIP can overlap with other pancreatic disorders, making the diagnosis more difficult.

## Conclusions

In conclusion, distinguishing between AIP and pancreatic cancer can be challenging due to their similarities. However, in this case, the lack of progression in the disease and normal liver function tests, combined with elevated serum IgG4 levels, helped rule out pancreatic cancer. Therefore, it is crucial to maintain a high level of suspicion and consider AIP as a possible diagnosis when investigating cases that mimic pancreatic cancer.
